# Use and satisfaction with key functions of a common commercial electronic health record: a survey of primary care providers

**DOI:** 10.1186/1472-6947-13-86

**Published:** 2013-08-09

**Authors:** Anil N Makam, Holly J Lanham, Kim Batchelor, Lipika Samal, Brett Moran, Temple Howell-Stampley, Lynne Kirk, Manjula Cherukuri, Noel Santini, Luci K Leykum, Ethan A Halm

**Affiliations:** 1 Division of General Internal Medicine, University of Texas Southwestern Medical Center, 5323 Harry Hines Blvd, Dallas, TX 75390, USA; 2 Division of Hospital Medicine, University of Texas Health Science Center at San Antonio, 7703 Floyd Curl Dr. MC7982, San Antonio, TX 78229, USA; 3 South Texas Veterans Health Care System, 7400 Merton Minter, San Antonio, TX 78229, USA; 4 Division of General Internal Medicine and Primary Care, Brigham and Women’s Hospital, 1620 Tremont St., OBC-3, Boston, MA 02120, USA; 5 Department of Family and Community Medicine, University of North Texas Health Science Center, 1650 S. Beach St, Fort Worth, TX 76105, USA; 6 Community Medicine Division, Parkland Health & Hospital System, 5201 Harry Hines Blvd, Dallas, TX 75235, USA

**Keywords:** Electronic health record (EHR), Attitude of health personnel, Attitude to computers, Primary care, Efficiency, Quality of care, Medical informatics/utilization

## Abstract

**Background:**

Despite considerable financial incentives for adoption, there is little evidence available about providers’ use and satisfaction with key functions of electronic health records (EHRs) that meet “meaningful use” criteria.

**Methods:**

We surveyed primary care providers (PCPs) in 11 general internal medicine and family medicine practices affiliated with 3 health systems in Texas about their use and satisfaction with performing common tasks (documentation, medication prescribing, preventive services, problem list) in the Epic EHR, a common commercial system. Most practices had greater than 5 years of experience with the Epic EHR. We used multivariate logistic regression to model predictors of being a structured documenter, defined as using electronic templates or prepopulated dot phrases to document at least two of the three note sections (history, physical, assessment and plan).

**Results:**

146 PCPs responded (70%). The majority used free text to document the history (51%) and assessment and plan (54%) and electronic templates to document the physical exam (57%). Half of PCPs were structured documenters (55%) with family medicine specialty (adjusted OR 3.3, 95% CI, 1.4-7.8) and years since graduation (nonlinear relationship with youngest and oldest having lowest probabilities) being significant predictors. Nearly half (43%) reported spending at least one extra hour beyond each scheduled half-day clinic completing EHR documentation. Three-quarters were satisfied with documenting completion of pneumococcal vaccinations and half were satisfied with documenting cancer screening (57% for breast, 45% for colorectal, and 46% for cervical). Fewer were satisfied with reminders for overdue pneumococcal vaccination (48%) and cancer screening (38% for breast, 37% for colorectal, and 31% for cervical). While most believed the problem list was helpful (70%) and kept an up-to-date list for their patients (68%), half thought they were unreliable and inaccurate (51%).

**Conclusions:**

Dissatisfaction with and suboptimal use of key functions of the EHR may mitigate the potential for EHR use to improve preventive health and chronic disease management. Future work should optimize use of key functions and improve providers’ time efficiency.

## Background

The federal government is investing $27 billion over ten years through the Health Information Technology for Economic and Clinical Health Act to increase the adoption and use of electronic health records (EHR) in the United States to improve health outcomes [1]. The use of EHRs has the potential to improve quality and safety in health care, but these improvements will only occur if providers understand and use key functions regularly and effectively [2]. The Office of the National Coordinator has defined a minimum list of EHR functionalities, known as “meaningful use” criteria, intended to promote the effective use of the EHR to drive improvements in safety, quality and efficiency [3]. “Meaningful use” requires a variety of EHR functions, ranging from visit documentation to clinical decision support [1]. Commercial EHR systems such as Epic are increasingly being adopted because their off-the-shelf capabilities meet meaningful use criteria required to qualify for financial incentives [4]. However, there is relatively little evidence available about providers’ use of and satisfaction with key functions, particularly in commercial EHRs.

One prior study found considerable variability in physician use of EHR functions. However, this study included physicians using different EHR systems in various stages of EHR implementation, making it challenging to attribute suboptimal use of specific functions to provider preference rather than to differences in the design and technology of the EHR, or inexperience with using the system [5,6]. Prior literature has also linked EHR use to high ratings of general satisfaction with the overall EHR system, medical practice, and career [7-9]. However, these studies did not examine providers’ satisfaction with specific key EHR functions, which may be important in understanding potential suboptimal use of embedded functions [10].

Therefore, the aims of this study were to determine: 1) how providers who practice in a mature EHR environment use key functions of a comprehensive commercial EHR system to perform common clinical tasks, with a particular interest in assessing use of structured documentation features which may improve efficiency and thoroughness; 2) their satisfaction with key functions, and 3) the time they spend on EHR activities outside of their clinic sessions.

## Methods

### Study population

We contacted all 210 adult primary care providers (PCPs) in eleven general internal medicine and family medicine practices affiliated with one of the following three health systems in Texas: University of Texas Southwestern, Parkland Health & Hospital System, and University of Texas Health Science Center at San Antonio. Most practices had greater than five years of experience using the Epic EHR (Epic Systems Corporation, Verona, WI), a common commercial EHR system.

This study was approved by the institutional review boards of the University of Texas Southwestern and the University of Texas Health Science Center at San Antonio, and included a waiver of the requirement for informed consent of participating providers.

### Epic EHR system

The Epic Ambulatory EHR is certified by the Office of the National Coordinator as a complete EHR system meeting “meaningful use” criteria, featuring computerized provider order entry, electronic prescribing, integrated clinical documentation, test result tracking, and active medication, allergy, and problem lists. The off-the-shelf system also includes basic clinical decision support tools, including a health maintenance module that creates automated plans for key preventive health measures and a drug-drug interaction pop-up alert. Among outpatient providers practicing in medium to large-sized clinics in the U.S. that currently have an EHR system, 26% use the Epic Ambulatory EHR [11].

### Survey

The survey was designed and refined by research team members with expertise in medical informatics, diffusion of innovation, and health services research. The questions included in the survey were guided by literature review of previous studies about providers’ use and satisfaction of the EHR [5-7,12,13]. We pilot tested the survey among a group of practicing physicians with experience using the Epic EHR for readability, clarity, and feedback. Items focused on how clinicians performed common tasks in the EHR (documentation, medication prescribing, health care maintenance/preventive services, problem list), how satisfied they were with these EHR features, and the time they spent on EHR tasks outside of the regular clinic session (See Additional file 1: Survey). We also asked how often PCPs used the electronic patient portal function (MyChart) to communicate with patients for the providers practicing in the two health systems that had this function activated. Providers self-reported data on demographics, specialty, years of experience using an EHR, and avidity for being an “early adopter” of information technologies in general [14].

We distributed a self-administered, web-based survey between August 2011 and December 2011. The survey was sent to PCPs by e-mail from his or her respective Division Chief or Medical Director with a link to the online survey and an incentive of entering a lottery upon completion to win a $100 gift certificate. Two reminder e-mails spanning a four week period were subsequently sent to non-respondents. In addition, clinical leaders and site-specific physician champions encouraged participation by in-person reminders during clinic team meetings.

### Statistical analysis

Descriptive data are expressed as percentages. Respondents and non-respondents were compared using chi-squared tests. We defined “structured documenters” as providers who reported using electronic prepopulated ‘dot phrases’ or electronic templates as the “usual” method of documenting for at least two of the three clinical note sections (history, physical exam, and assessment and plan). ‘Dot phrases’ are electronic shorthand for longer phrases or structured data (i.e. “.med” can import the whole medicine list and “.vitals” the full vital signs). We modeled predictors of being a structured documenter using logistic regression with a stepwise backward elimination algorithm, iteratively removing candidate predictors with a p-value > 0.2. Candidate predictors thought to influence documentation style were chosen based on expert opinion and literature review, including the Unified Theory of User Acceptance of Technology and diffusion of innovation [10,14,15]. The predictor, years since graduation, violated the assumption of linearity and was modeled using restricted cubic splines [16]. The final model included early adopter status, years since graduation, and specialty, accounting for clustering at the clinic level. We performed model diagnostic tests, including Hosmer-Lemeshow goodness of fit.

The data were analyzed using STATA statistical software, version 12.0 (Stata-Corp, College Station, Texas).

## Results

Of the 210 providers sent surveys, 146 responded yielding a response rate of 70%. The majority of PCPs who responded were female (61%), attending physicians (64%), had graduated from medical school or the equivalent less than 20 years ago (59%), and self-classified themselves as early-adopters of new technology (62%). About half of the providers (53%) had at least three years of experience using an EHR system and only 4% had less than one year of experience (Table 1). Non-respondents were similar to respondents with respect to gender and specialty, but were more likely to be residents than attending physicians (p value = .02). Overall, respondents reported a high level of use and satisfaction with EHR functions facilitating transactional tasks such as electronic prescribing. There was a wide degree of variability in use and satisfaction with functions aimed at facilitating medical decision-making, including clinical documentation, health maintenance and preventive screening, problem list updating and electronic messaging.

**Table 1:** Characteristics of Primary Care Provider Respondents (N = 146)

**Characteristic**	**N (%)**
Male	55 (38%)
Specialty	
Internal medicine	110 (75%)
Family medicine	36 (25%)
Position	
Attending	93 (64%)
Resident	37 (25%)
Midlevel provider*	15 (10%)
Number of Years since Graduation	
1-9	47 (32%)
10-19	39 (27%)
21-29	23 (16%)
≥ 30	19 (13%)
≥ 3 years prior EHR experience	78 (53%)
Early adopter of technology	90 (62%)

*Abbreviations*: EHR, electronic health record.

*Nurse practitioner or physician assistant.

### Providers’ use and satisfaction with specific EHR functions

#### Encounter note

**Figure 1:**
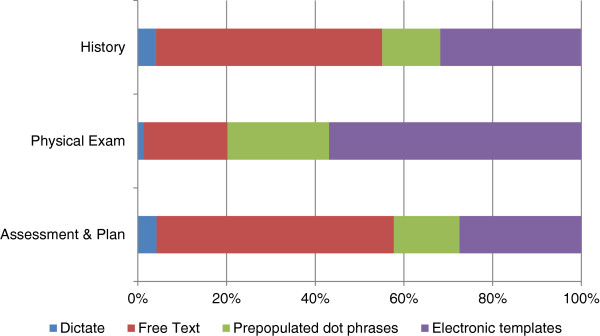
Methods PCPs use to document sections of the clinical note in the EHR.

Figure 1 shows that the most providers used different methods for documentation in the EHR for different sections of the outpatient encounter note. About half of PCPs “usually document” the history (51%) and the assessment and plan (54%) sections of the note by typing free text. In contrast, most (57%) used standardized templates to document the physical exam. The second most common strategy employed was templates for the history and assessment and plan, and pre-populated ‘dot phrases’ for the physical exam. Very few used dictation as a means of clinical documentation despite it being available and supported by the health information management department. Only one-third of providers (36%) used the same documentation style throughout the entire note.

**Figure 2:**
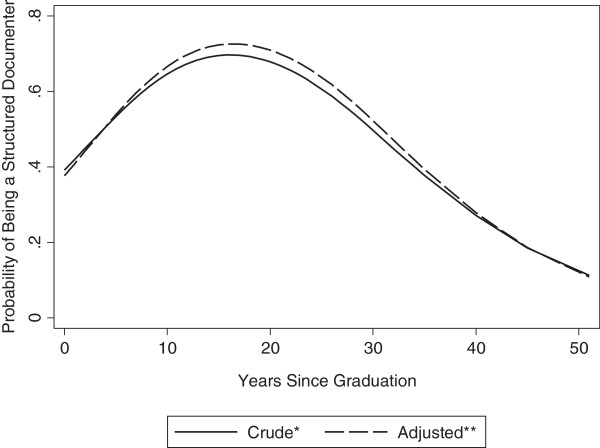
**Relationship between the Probability of a Provider Being a Structured Documenter and Years since Graduation.** Structured documenters were defined as providers who reported using electronic prepopulated ‘dot phrases’ or electronic templates as the “usual” method of documenting for at least two of the three clinical note sections (history, physical exam, and assessment and plan). *Modeled using restricted cubic splines, accounting for clustering at the clinic level. **Adjusted for early adopter of new information technology status and provider specialty.

About half of the providers (55%) were structured documenters—using electronic ‘dot phrases’ or templates to document at least two sections in the clinical note. Family medicine providers had 3.3 times the odds (95% CI, 1.4-7.8, p = .007) of being a structured documenter compared to internal medicine providers (Additional file 2: Table S1). The association between years since graduation and being a structured documenter was more nuanced (Figure 2). There was a near linear increase in the probability of being a structured documenter up until about 14 years after graduation, but thereafter the probability decreases exponentially with very low probabilities for those providers who are greater than 30 years out from graduation.

#### Health care maintenance and preventive services

Since one anticipated benefit of the EHR is to improve adherence with preventive care guidelines, we asked providers how well the EHR performed in this domain. By and large, the results were mixed and reflected a heterogeneous experience with attempting to document these activities. Almost three-quarters of providers were satisfied with the ability to document completion of a pneumococcal vaccine (72%) in the EHR, while only about half were satisfied with options for documenting completion of screening tests for cancer (57% for breast, 45% for colorectal, and 46% for cervical).

Documentation practices for preventive health measures performed outside of the health system were varied. Three-quarters of providers (72%) consistently documented pneumococcal vaccine performed elsewhere in a single location in the EHR. Among these providers, half used the historical immunization section (50%), one-third free texted in the note (33%), and a fraction used the health maintenance module (16%). Among the two-thirds of providers (66%) who documented the results of mammography performed outside the health system in a consistent location, 44% free texted in the note, 15% used the past medical history field, and only 32% used the health maintenance section, which is the recommended process.

With regard to decision support to promote preventive care, fewer than half of providers (48%) were satisfied with EHR reminders for overdue pneumococcal vaccine and only about a third of PCPs were satisfied with the EHR’s ability to remind them about overdue cancer screening (38% for breast, 37% for colorectal, and 31% for cervical).

Overall, PCPs’ opinion of the design and function of the health maintenance module was divided, with 35% dissatisfied, 29% with no opinion, and 35% satisfied.

#### Medication prescribing

Preferred medication prescribing styles were less varied, with most PCPs electronically prescribing new (79%) and refill (90%) medications compared with phoning in or printing out on paper. Opinions about the EHRs’ drug-drug interaction pop-up alerts (which are hard stops) were mixed with 61% of PCPs expressing satisfaction but 28% being dissatisfied with it.

#### Problem list

The problem list in the Epic EHR allows providers to update and view diagnostic and therapeutic plans linked to specific problems. The clinics encourage providers to maintain an active, accurate problem list to be the source of ‘truth’ in the patient’s electronic record. The majority of providers agreed that the problem list was helpful (70%), accepted personal responsibility for keeping the problem list up-to-date (73%), and reported actually keeping an up-to-date and accurate list for their patients (68%). However, half of the providers (51%) believed the problem list was unreliable and inaccurate and a majority erroneously believed that adding an encounter diagnosis automatically updates the problem list (60%).

#### Electronic messaging

Among the 59 providers who worked in health systems with an electronic patient portal in their EHR, 69% used it to communicate with patients some or more of the time.

### Time spent using the EHR

**Figure 3:**
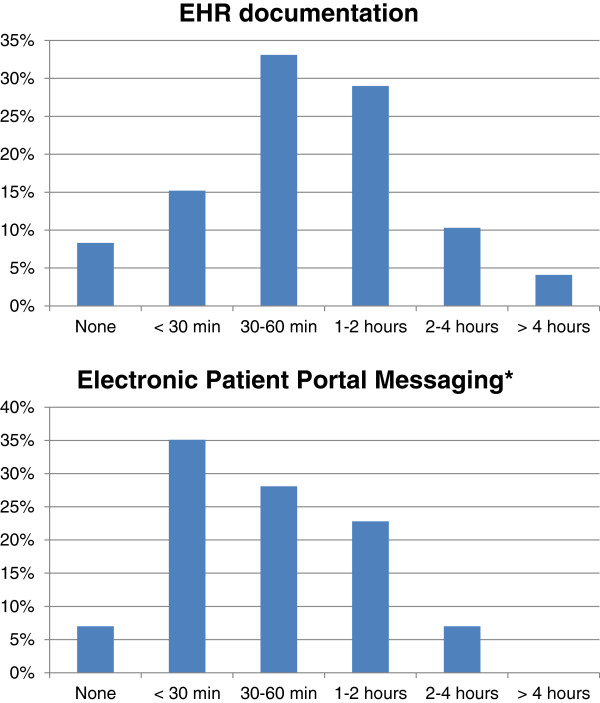
**Extra Time PCPs Spent Completing EHR documentation and Responding to Electronic Messages after Each Half-Day Clinic Session.** *Data from 59 providers who had access to the electronic patient portal function (MyChart).

Figure 3 shows that providers spend a considerable amount of time using the EHR outside of their scheduled clinic time. Nearly half of the providers (43%) reported spending one or more extra hours beyond each scheduled half-day clinic session completing EHR documentation, and 30% reported spending at least one extra hour responding to electronic messages from patients (MyChart) in the EHR per half-day clinic session.

## Discussion

Among a diverse group of PCPs practicing in clinics experienced with using a common commercial EHR system, we identified variability in providers’ use and satisfaction with many key EHR functions. A majority of providers were not regularly using or were not highly satisfied with a few core features of the EHR, including documentation of preventive services and the health maintenance module.

There was great variability in the extent to which users availed themselves of more structured electronic note writing tools (like templates) and most used a ‘mix and match’ approach of free text for the history and assessment, and templates for the exam. Whether this is because the exam is the easiest part of the note to template, or there is lack of awareness of or low opinions about the user friendliness of other EHR note writing functions is not known. It should be noted that the EHR encourages some “free text” writing for history and assessments and plan sections even within templates.

About half of the providers in our sample routinely used structured EHR documentation. While it remains uncertain whether there is a single best method to document notes, the use of structured documentation has been associated with improved performance on several quality measures compared to those using free text or dictating [12]. Additionally, using structured documentation such as templates (as opposed to free texting the note from scratch) could potentially be a time-saving benefit of the EHR. On the contrary, structured documentation may promote bloated notes with redundant or irrelevant information and cloning, the practice of copying and pasting past documentation into the current encounter note. Our findings suggest there is a generational influence on structured EHR documentation use, with both extremes of age having the lowest probabilities. For the oldest providers it may be related to a combination of a lack of awareness, training, or interest in engaging with the EHR. In contrast, the youngest providers may have unmet expectations for technological innovations in the EHR and as a result, may shy away from relatively inflexible templates with suboptimal user interfaces [17]. Family medicine providers were more likely to use structured documentation compared to their internal medicine counterparts, and may be due to differences in the way different specialties interact with the EHR.

While the EHR has been promoted as a useful tool to improve preventive health, there is mixed evidence for the effectiveness of EHR-based clinical decision support (CDS) reminders for improving cancer screening and vaccination rates [18]. In addition, prior research has not found an association between use of an EHR and improved performance on these process measures for health prevention [19-22]. We found that providers reported substantial variability in documentation practices for preventive services (cancer screening and vaccinations) and expressed considerable dissatisfaction with the structure and function of the existing health care maintenance module and its associated reminder system, which may have repercussions for EHR-based strategies to improve preventive health.

Our findings highlight a potential explanation for why the use of EHRs and CDS tools may be ineffective in improving concordance with delivery of recommended preventive care. CDS rules typically use results from structured data fields to make inferences about when certain preventive health measures are due. If providers are not consistently documenting results in structured fields in the EHR, such as the health maintenance module and the historical immunization section, then CDS tools will not have the necessary data to provide accurate and timely recommendations for providers.

There has been increased attention directed towards the ‘problem list’ as a key focus of meaningful use and driver of CDS. Our findings highlight some of the challenges that remain, because while most PCPs thought the problem list was important and kept it up-to-date, most erroneously thought listing a diagnosis in the ‘encounter’ section automatically carried over to the problem list, and half worried that the problem list was unreliable and inaccurate. This suggests that quality measurement and CDS strategies that rely solely on diagnoses in the problem list may be inaccurate. It also suggests that in order for the problem list to help promote meaningful use and be an effective driver of CDS more attention needs to be paid to understanding and allaying providers’ concerns on this issue.

In addition to quality of care, another important outcome to evaluate the success of EHRs is efficiency. Previous time and motion studies of providers using the EHR compared to paper charts found that time-efficiency was not likely to be achieved during the clinic visit with use of an EHR [23,24]. Our study suggests that using an EHR can be considerably time-consuming for many providers even after the scheduled clinic session. Extrapolating our findings to full-time PCPs practicing nine half-day clinic sessions a week, nearly half of providers are spending greater than 9 extra hours a week beyond their scheduled clinic sessions completing documentation in the EHR and a third are spending at least 9 extra hours a week responding to electronic messages. This is a substantial after-hour “time tax” on providers who are already pressured to see more patients in light of waning reimbursements and may contribute to the high burnout risk seen in primary care [25].

Our study has several important limitations. First, though our sample was large and diverse, we surveyed PCPs using a single commercial EHR, so the generalizability to other systems besides Epic is unknown. We intentionally surveyed providers using a single system to better understand the use of key functions independent of differences in EHR design. In addition, Epic is considered to be a ‘best-of-breed’ comprehensive system with a dominant market share, accounting for one-quarter of providers practicing in medium to large-sized clinics that currently use an EHR system [11]. Therefore, lessons learned from this study may thus be readily applicable to a large proportion of primary care providers. In addition, many of the Epic features we asked about are similar to those in most commercial and home-grown EHRs, so our findings may have implications for the larger EHR community. Second, this study relied on providers’ self-reported use of the EHR. We attempted to mitigate possible recall bias by asking providers about their “typical” experiences rather than asking them to report their specific use patterns for previous clinic sessions. The providers included in this survey were all current users of the EHR in their daily clinical practices, helping to guard against inaccuracies in respondents’ historical accounts of their engagement with the system. Third, we used a web-based survey to ask providers about their experiences using an EHR system. This approach may have selected participants who were more likely to be technology-savvy and potentially use more advanced features of the EHR. However, our findings of suboptimal use and dissatisfaction for several key functions suggest that if we selected a greater number of EHR enthusiasts then we may have underestimated the perceived deficiencies of the EHR. In addition, we found that two-thirds of providers self-classified as early adopters of technology, a rate nearly identical in a study of providers by Kim et al. that employed both web-based and paper questionnaires [26].

## Conclusion

The results of this study suggest that implementing a full-featured EHR that meets “meaningful use” criteria is insufficient and that we should shift our attention from adopting an EHR to optimizing provider use of key functions and improving providers’ time efficiency. Potential solutions to extract maximum benefit from the EHR include: refinement of EHRs to be more intuitive and user-friendly especially for common clinical tasks, development of more intelligent, clinically nuanced CDS, more effective provider training on use of advanced EHR functionalities, and greater attention to how to best use the EHR to improve the clinical workflow efficiency.

This study points to a sizeable gap between the ‘dream’ of policymakers’ vision for EHRs and the ‘reality’ of frontline PCPs’ current use of an industry-leading commercial system. The availability of certain functions that constitute “meaningful use” may be necessary but insufficient to ensure ‘meaningfully’ effective use of EHRs. Provider dissatisfaction with and suboptimal use of more advanced features of the EHR may in part explain the lack of association between EHR use and improved quality of care [19-22]. Further research is needed to both understand the proximal factors creating the gap between availability of functions and suboptimal use and how best to narrow the divide to improve quality and efficiency.

## Abbreviations

PCPs: Primary care providers; EHR: Electronic health record.

## Competing interests

The authors declare that they have no competing interests.

## Authors’ contributions

All authors in this study have been involved in concept and design, critical revision of the manuscript for important intellectual content, and giving final approval of the manuscript for submission. AM, EH, HL and LS designed the study and survey. AM performed data analysis and interpretation and drafting of the manuscript, with significant contributions from EH, HL, and BM. BM, THS, LK, MC, NS, LL, and KB participated in refinement and implementation of the study and review of the results and manuscript.

## Pre-publication history

The pre-publication history for this paper can be accessed here:

http://www.biomedcentral.com/1472-6947/13/86/prepub

## Supplementary Material

Additional file 1 Survey. Click here for file

Additional file 2: Table S1 Predictors of a Provider Being a Structured Documenter*. Click here for file
