# Supplementary material for: Use and satisfaction with key functions of a common commercial electronic health record: a survey of primary care providers
Source: BMC Med Inform Decis Mak. 2013 Aug 9;13:86. doi: 10.1186/1472-6947-13-86 (PMC3750656; doi:10.1186/1472-6947-13-86)
Supplement: Additional file 1 — Survey. [file 1472-6947-13-86-S1.pdf]

We are conducting a research project to better understand how you use the EMR and your opinions about its various features. The focus of this survey is the use of the EMR in your usual OUTPATIENT clinical setting.

1. How long have you been using electronic medical records (EPIC or otherwise)?

- [ ] < 6 months
- [ ] 6-12 months
- [ ] 1-2 years
- [ ] 3-5 years
- [ ] > 5 years

2. How do you usually document the following in the medical record?

	Dictate	Type free text note	Type note using SmartPhrases (eg .med to import medications)	Type into template subsection (eg. SmartText, Notewriter)
History	☐	☐	☐	☐
Physical Exam	☐	☐	☐	☐
Assessment and Plan	☐	☐	☐	☐

3. How do you or your staff usually prescribe the following?

	Print on paper	Call-in	E-fax	E-prescription
New medications	☐	☐	☐	☐
Refill medications	☐	☐	☐	☐

4. For each ½ day clinic session, how much time beyond the scheduled session do you spend on the following activities?

	0 minutes, I do all my charting during the session	< 30 minutes extra	30-60 minutes extra	1-2 hours extra	2-4 hours extra	> 4 hours extra
EMR documentation	☐	☐	☐	☐	☐	☐
MyChart messages	☐	☐	☐	☐	☐	☐

5. Where would you document the results of a mammography test completed elsewhere? Please check all that apply.

- ☐ Free text in note
- ☐ Health maintenance section / health maintenance module
- ☐ Surgical or Medical history

Other (please specify)

6. Where would you document a pneumococcal vaccine given elsewhere? Please check all that apply

- [ ] Free text in note
- [ ] Health maintenance section / health maintenance module
- [ ] Historical immunization records

Other (please specify)

Current Satisfaction with the EMR

Questions #7-10 are about your satisfaction with the EMR currently at handling the following.

7. Documenting the following preventative services when completed

	Very satisfied	Satisfied	No opinion	Dissatisfied	Very dissatisfied
Pneumonia vaccination	☐	☐	☐	☐	☐
Mammography	☐	☐	☐	☐	☐
Colorectal cancer screening	☐	☐	☐	☐	☐
Cervical cancer screening	☐	☐	☐	☐	☐

8. Reminding me when the following preventative services are due

	Very satisfied	Satisfied	No opinion	Dissatisfied	Very dissatisfied
Pneumococcal vaccine	☐	☐	☐	☐	☐
Mammography	☐	☐	☐	☐	☐
Colorectal cancer screening	☐	☐	☐	☐	☐
Cervical cancer screening	☐	☐	☐	☐	☐

9. The EMR drug-drug interaction pop-up alerts

☐ Very satisfied ☐ Satisfied ☐ No opinion ☐ Dissatisfied ☐ Very dissatisfied ☐ Not applicable

10. The health maintenance section / health maintenance module

☐ Very satisfied ☐ Satisfied ☐ No opinion ☐ Dissatisfied ☐ Very dissatisfied ☐ Not applicable

Please indicate the extent to which you agree or disagree with each of the following statements.

11. The problem list in the EMR is helpful for patient care.

☐ Strongly Agree ☐ Agree ☐ No Opinion ☐ Disagree ☐ Strongly Disagree

12. Keeping the problem list up to date is my responsibility.

☐ Strongly Agree ☐ Agree ☐ No Opinion ☐ Disagree ☐ Strongly Disagree

13. I keep an up to date and accurate problem list for each of my patients.

☐ Strongly Agree ☐ Agree ☐ No Opinion ☐ Disagree ☐ Strongly Disagree

14. Adding an encounter diagnosis automatically updates the problem list.

☐ Strongly Agree ☐ Agree ☐ No Opinion ☐ Disagree ☐ Strongly Disagree

15. The problem list is unreliable and inaccurate.

☐ Strongly Agree ☐ Agree ☐ No Opinion ☐ Disagree ☐ Strongly Disagree

Please indicate the extent to which you agree or disagree with each of the following statements.

16. If I heard about a new information technology, I would look for ways to experiment with it.

☐ Strongly Agree ☐ Agree ☐ No Opinion ☐ Disagree ☐ Strongly Disagree

17. Among my peers, I am usually the first to try out new information technologies.

☐ Strongly Agree ☐ Agree ☐ No Opinion ☐ Disagree ☐ Strongly Disagree

18. In general, I am hesitant to try out new information technologies.

☐ Strongly Agree ☐ Agree ☐ No Opinion ☐ Disagree ☐ Strongly Disagree

19. I like to experiment with new information technologies.

☐ Strongly Agree ☐ Agree ☐ No Opinion ☐ Disagree ☐ Strongly Disagree

Physician Demographics

20. What is your gender?

- [ ] Male
- [ ] Female

21. When did you graduate from medical school?

22. What is your current position?

- [ ] Attending physician
- [ ] Fellow
- [ ] PGY-1
- [ ] PGY-2
- [ ] PGY-3
- [ ] NP/PA
