# Supplementary material for: Use and satisfaction with key functions of a common commercial electronic health record: a survey of primary care providers
Source: BMC Med Inform Decis Mak. 2013 Aug 9;13:86. doi: 10.1186/1472-6947-13-86 (PMC3750656; doi:10.1186/1472-6947-13-86)
Supplement: Additional file 2: Table S1 — Predictors of a Provider Being a Structured Documenter*. [file 1472-6947-13-86-S2.docx]

| **Appendix Table – Predictors of a Provider Being a Structured Documenter*** | | | |
| --- | --- | --- | --- |
| **Candidate Predictors** | Univariate OR  (95% CI) | Multivariate OR  (95% CI)** | Adjusted p-value |
| Years since graduation^†^ |  |  |  |
| Spline term 1 | 1.12 (1.01-1.25) | 1.14 (1.06-1.23) | .001 |
| Spline term 2 | 0.84 (0.74-0.96) | 0.83 (0.73-0.93) | .002 |
| Early adopter of technology |  |  |  |
| No | Ref | Ref |  |
| Yes | 1.25 (0.82-1.90) | 1.38 (0.89-2.13) | .15 |
| Specialty |  |  |  |
| Internal medicine | Ref | Ref |  |
| Family medicine | 2.55 (1.01-6.43) | 3.28 (1.38-7.81) | .007 |
| Experience using an EHR |  |  |  |
| < 3 years | Ref | - | - |
| ≥ 3 years | 1.25 (0.74-2.13) |  |  |
| Position |  | - | - |
| Attending | Ref |  |  |
| Resident | 0.55 (0.15-1.96) |  |  |
| Midlevel | 1.61 (0.67-3.90) |  |  |
| * Structured documenters were defined as providers who reported using electronic prepopulated ‘dot phrases’ or electronic templates as the “usual” method of documenting for at least two of the three clinical note sections (history, physical exam, and assessment and plan)  ** Multivariate model was selected using a stepwise backward elimination algorithm, iteratively removing candidate predictors with a p-value > 0.2  ^†^ Years since graduation was modeled using restricted cubic splines with knots at 1, 14, and 32 years | | | |
